# Case report: Hilar metastasis of breast cancer: A single-center retrospective case-control study

**DOI:** 10.3389/fsurg.2023.1025287

**Published:** 2023-02-21

**Authors:** Ruohan Yang, Lin Jia, Zheng Lv, Jiuwei Cui

**Affiliations:** Cancer Center, the First Hospital of Jilin University, Changchun, China

**Keywords:** breast cancer, hilar metastasis, peripheral lung metastasis, overall survival, prognosis

## Abstract

**Purpose:**

The lungs are a common metastatic organ in breast cancer, mainly due to blood metastasis. On imaging, most metastatic lesions show a peripheral round mass in the lung, occasionally with a hilar mass as the primary manifestation, showing burr and lobulation signs. This study aimed to investigate breast cancer patient's clinical characteristics and prognosis with two different metastatic sites in the lung.

**Methods:**

We retrospectively analyzed patients admitted to the First Hospital of Jilin University between 2016 and 2021 diagnosed with breast cancer lung metastases. Forty breast cancer patients with hilar metastases (HM) and 40 patients with peripheral lung metastases (PLM) were matched 1:1 using a pairing method. To analyze the patient's prognosis, the clinical characteristics of patients with two different metastatic sites were compared using the chi-square test, Kaplan–Meier curve, and Cox proportional hazards model.

**Results:**

The median follow-up time was 38 months (2–91 months). The median age of patients with HM was 56 years (25–75 years), and that of patients with PLM was 59 years (44–82 years). The median overall survival (mOS) was 27 months in the HM group and 42 months in the PLM group (*p* = 0.001). The results of the Cox proportional hazards model showed that the histological grade (hazard ratio = 2.741, 95% confidence interval 1.442–5.208, *p* = 0.002) was a prognostic factor in the HM group.

**Conclusion:**

The number of young patients in the HM group was higher than that in the PLM group, with higher Ki-67 indexes and histological grades. Most patients had mediastinal lymph node metastasis, with shorter DFI and OS and poor prognosis.

## Introduction

According to the report of Global Cancer in 2020, breast cancer has become a malignant tumor with the highest incidence. There are estimated to be 2.3 million newly diagnosed cases in 2020 (1). Even with conventional treatment, approximately 40% of patients develop distant metastases 5 years post-surgery (2, 3). The lung is the second most common distant metastatic target site in breast cancer (4, 5). Cancer cells typically infiltrate surrounding tissues through the blood circulation system and colonize the lung, followed by gradual accumulation to form clinically detectable overt lung metastatic lesions (6, 7). Lung metastases of breast cancer have the following characteristics on lung computed tomography (CT) imaging: (1) multiple hematogenous lung metastases: breast cancer cells invade the capillaries reaching the lungs through blood circulation, forming multiple, high-density, well-defined round-like masses in the periphery of the lungs, (2) endobronchial metastases present as a single spiky mass in the hilum similar to that found in central lung cancer, (3) airspace metastasis: tumor cells spread along the intact alveolar wall, (4) lymphatic metastasis, and (5) solitary pulmonary nodules. Among these manifestations, hematogenous multiple lung metastases are the most common (8, 9). However, pulmonary metastases with hilar masses as the primary manifestation are rare in clinical practice (10) and include masses with a spiky sign, obstructive pneumonia, and mediastinal lymphadenopathy (11, 12). Previous studies have found that bronchoscopy in patients with HM often reveals an endobronchial mass accompanied by thickening of the bronchial wall, which becomes a polypoid growth (irregular round solid mass). A small number of cases may also show only partial surrounding mucosal edema. And there were no raised lesions because of the tumor's continuous infiltration of the bronchial mucosal wall. Since the lesions are diffuse bronchial submucosal metastases, they are not easy to detect on routine examination; however, the patient's symptoms of dry cough persisted, and breast cancer metastasis was confirmed through bronchoscopy biopsy (13–15). Some studies consider metastatic lesions in the hilum as bronchopulmonary metastasis (16). The incidence of bronchopulmonary metastasis is the highest among breast, colon, and kidney cancers (17, 18). Clinical manifestations include cough, chest tightness, and hemoptysis. Pathological diagnosis is the gold standard for confirming the origin of metastases (19). Patients with HM have a worse prognosis than those with metastases at other sites. Kim et al. reported that the longest overall survival (OS) was 58 months, the shortest OS was 1 month, and the median OS (mOS) was 15 months (14). There are few studies on the clinical characteristics and prognosis of breast cancer patients with HM, all of which are case reports and lack a large sample size.

We conducted a retrospective case-control study in combination with cases from our center to further analyze the clinical characteristics and prognosis of patients with hilar and PLM breast cancers.

## Methods

### Study population

Eighty patients with pathologically diagnosed breast cancer who visited the Cancer Center of the First Hospital of Jilin University between January 2016 and January 2021 were selected as research participants. The inclusion criteria were as follows: (1) patients with complete medical records, (2) and patients with HM who had undergone either bronchoscopy or pathological lung tissue biopsy to confirm the breast cancer metastases.

According to the inclusion and exclusion criteria, the 80 study participants were divided into two groups: 40 patients in the HM group and 40 in the 1:1 matched PLM group. All patients were not treated surgically after the diagnosis of metastatic disease, and all were treated with chemotherapy to control disease progression. Patients were followed up in our outpatient clinic after every two cycles of chemotherapy, and lung CT examinations were performed to assess disease progression.

The primary outcomes were defined as follows.

Overall survival (OS): The time from the occurrence of lung metastasis to the death of patients.

Progression-free survival (PFS): The time from the occurrence of lung metastasis to tumor progression or death from any cause.

Disease-free interval (DFI): time from the end of treatment to recurrence at any site.

### Statistical analysis

A retrospective analysis was used to compare the clinical characteristics of the patients, and the chi-square test or Fisher's exact test was used to compare categorical data between the groups. Overall survival curves were plotted using the Kaplan–Meier method, and differences between survival curves were tested using the log-rank test. After screening for PFS-related risk factors using the univariate Cox regression method (*p* < 0.05), a multivariate Cox regression model was fitted to determine independent prognostic factors.

OS and PFS curves were plotted using the Kaplan–Meier method for patients with lung metastases at the first metastatic site. Differences between survival curves were tested using the log-rank test.

Statistical significance was defined as a two-sided *p*-value of <0.05. All statistical tests were performed using SPSS 23.0 (IBM Corporation Released 2013. IBM SPSS Statistics for Windows, Version 23.0, Armonk, NY).

## Results

### Clinical characteristics of patients in HM and PLM groups

Of the 80 patients in this study, 40 developed HM, 29 of whom had a hilar mass as the first metastatic site. Forty patients had peripheral pulmonary metastasis, 24 of whom had the lungs as the first metastatic site. All patients were women and denied a family history of breast cancer, and 59 were postmenopausal. The median follow-up duration was 38 months (ranging from 2 to 91 months). Our study found that the median age of patients in the HM group was 56 years (with a range of 25–75 years), and the median age of patients in the PLM group was 59 years (with a range of 44–82 years). The age of patients with the hilar region as the first metastatic site was 55 years (with a range of 25–73 years), and the age of patients with the lung as the first metastatic site was 58 years (with a range of 44–74 years). We analyzed multiple clinical features, such as molecular typing and histological grade, in the two groups. The specific results are detailed in Tables 1, 2.

**Table 1:** Clinical characteristics of the two groups of patients.

	Hilar metastases group	Peripheral lung metastases group group	*p* value
**Age**			
**Median age (range)**	56 (25–75)	59 (44–82)	
≤40	9 (22.5%)	0 (0)	
>40	31 (77.5%)	40 (100%)	0.002
**Menstrual condition**			
Menopause	31 (77.5%)	28 (70%)	
Non-menopausal	9 (22.5%)	12 (30%)	0.46
**Molecular typing**			
Luminal A	17 (42.5%)	21 (52.5%)	
Luminal B	10 (25%)	6 (15%)	
HER-2 overexpression	6 (15%)	9 (22.5%)	
Triple negative	7 (17.5%)	4 (10%)	0.45
**Ki-67 expression**			
<15%	4 (10%)	14 (35%)	
≥15%	36 (90%)	26 (65%)	0.007
**Histological grading**			
Grade I	6 (15%)	16 (40%)	
Grade II	15 (37.5%)	14 (35%)	
Grade III	19 (47.5%)	10 (25%)	0.025
**Mediastinal lymph node metastasis**			
Yes	24 (60%)	10 (25%)	
No	16 (40%)	30 (75%)	0.002
**First metastatic site**			
Lung	29 (72.5%)	24 (60%)	
Liver	2 (5%)	6 (15%)	
Bone	4 (10%)	3 (7.5%)	
Brain	3 (7.5%)	4 (10%)	
Other	2 (5%)	3 (7.5%)	0.565
**Whether the first episode is visceral metastasis**			
Yes	34 (85%)	28 (70%)	
No	6 (15%)	12 (30%)	0.09
**Disease-free interval (DFI)**			
≤12 months	21 (52.5%)	10 (25%)	
>12 months	19 (47.5%)	30 (75%)	0.012

**Table 2:** Comparison of clinical characteristics between patients with the hilar region and lung as the first metastatic sites.

	Hilar metastasis (*n* = 29)	Lung metastasis (*n* = 24)	*p* value
**Age**			
**Median age (range)**	55 (29–73)	58 (44–74)	
≤40	3 (22.5%)	0 (0)	
>40	26 (77.5%)	24 (100%)	0.105
**Menstrual condition**			
Menopause	20 (77.5%)	19 (70%)	
Non-menopausal	9 (22.5%)	5 (30%)	0.40
**Molecular typing**			
Luminal A	13 (42.5%)	12 (52.5%)	
Luminal B	7 (25%)	3 (15%)	
HER-2 overexpression	3 (15%)	4 (22.5%)	
Triple negative	6 (17.5%)	5 (10%)	0.70
**Ki-67 expression**			
<15%	4 (13.8%)	18 (75%)	
≥15%	25 (86.2%)	6 (25%)	0.001
**Histological grading**			
Grade I	5 (17.2%)	10 (41.7%)	
Grade II	10 (34.5%)	10 (41.7%)	
Grade III	14 (48.3%)	4 (16.6%)	0.006
**Mediastinal lymph node metastasis**			
Yes	11 (37.9%)	7 (29.2%)	
No	18 (62.1%)	17 (70.8%)	0.502

The clinical manifestations of patients in the HM group were irritable cough, chest distress, and dyspnea. Most patients in the PLM group had no typical clinical manifestations. A summary of clinical manifestations in the two groups is shown in Table 3.

**Table 3:** Clinical manifestations of the two groups of patients.

	Hilar metastases group	Peripheral lung metastases group
Irritating dry cough	14 (35%)	5 (12.5%)
Chest tightness	5 (12.5%)	2 (5%)
Shortness of breath	4 (10%)	1 (2.5%)
Asymptomatic	17 (42.5%)	32 (80%)

**Figure 1:**
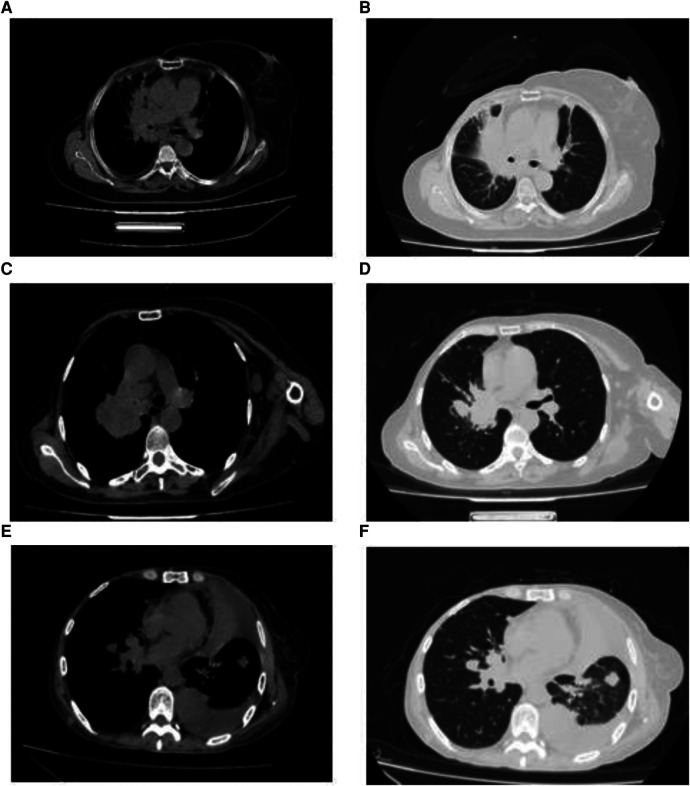
Imaging features of breast cancer patients with hilar metastasis (**A–F**): lung computed tomography images of three patients showed a single hilar mass with mediastinal lymphadenopathy.

Most patients in the HM group showed a single lesion in the hilum, accompanied by obstructive pneumonia and mediastinal lymphadenopathy on imaging. On imaging, patients in the PLM group showed multiple well-defined, round nodular shadows around the lung field. The two groups’ specific lung CT imaging characteristics are shown in Table 4. HM group showed imaging as a single mass with burr or lobar signs with obstructive pneumonia (*p* < 0.05). Figure 1 presents the lung CT images of patients in the HM group.

**Table 4:** Imaging features of the two groups of patients.

	Hilar metastases group	Peripheral lung metastases group	*p* value
Number of lung metastases			
Single	27 (67.5%)	0 (0)	
Multiple	13 (32.5%)	40 (100%)	<0.001
Burr or lobulation sign			
Yes	30 (75%)	4 (10%)	
No	10 (25%)	36 (90%)	<0.001
Obstructive pneumonia			
Yes	8 (20%)	1 (2.5%)	
No	32 (80%)	39 (97.5)	0.005
Mediastinal lymph node metastasis			
Yes	24 (60%)	10 (25%)	
No	16 (40%)	30 (75%)	0.12

### Prognosis

**Figure 2:**
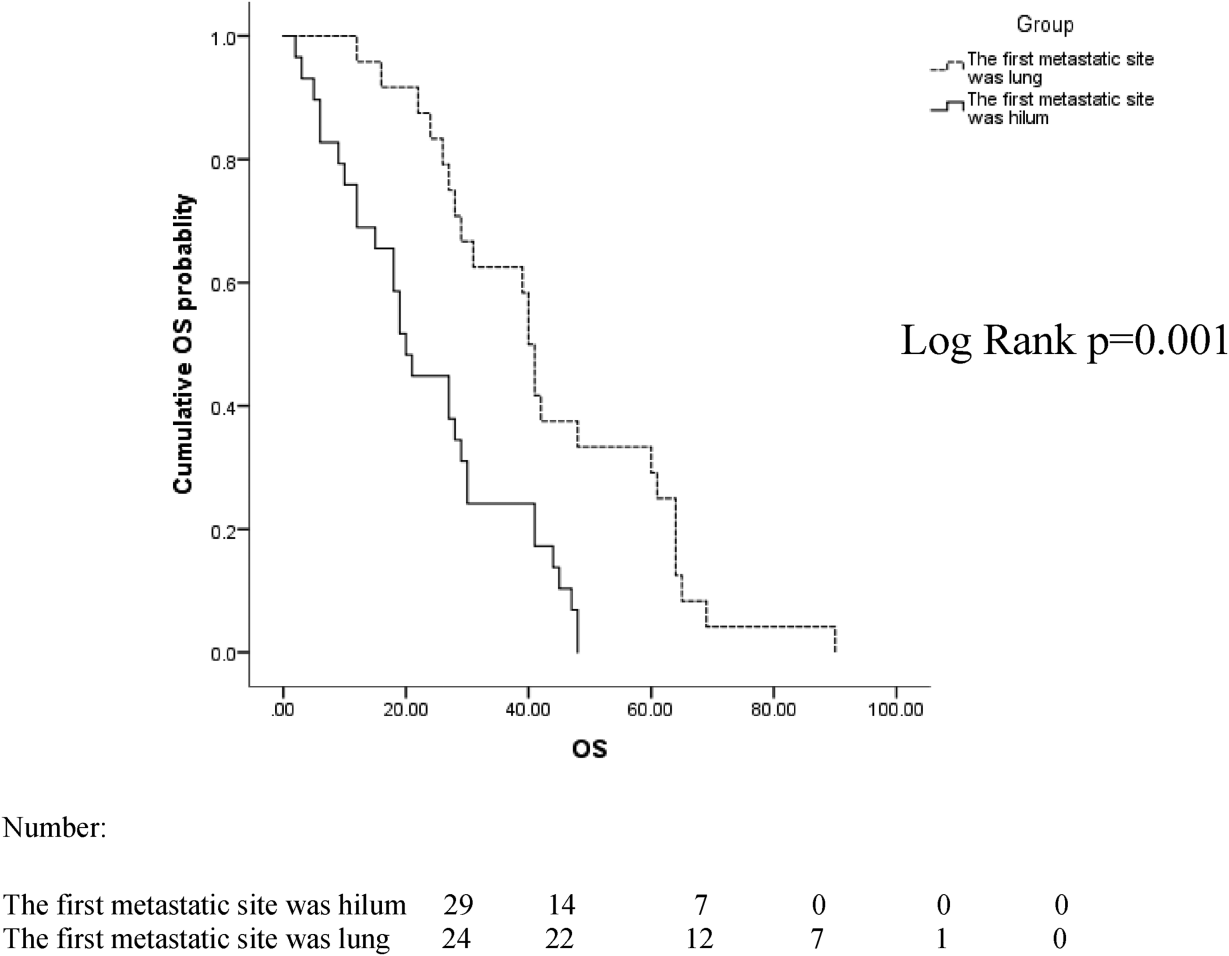
Overall survival (OS) curves of patients with lung and hilar regions as the first metastatic sites.

**Figure 3:**
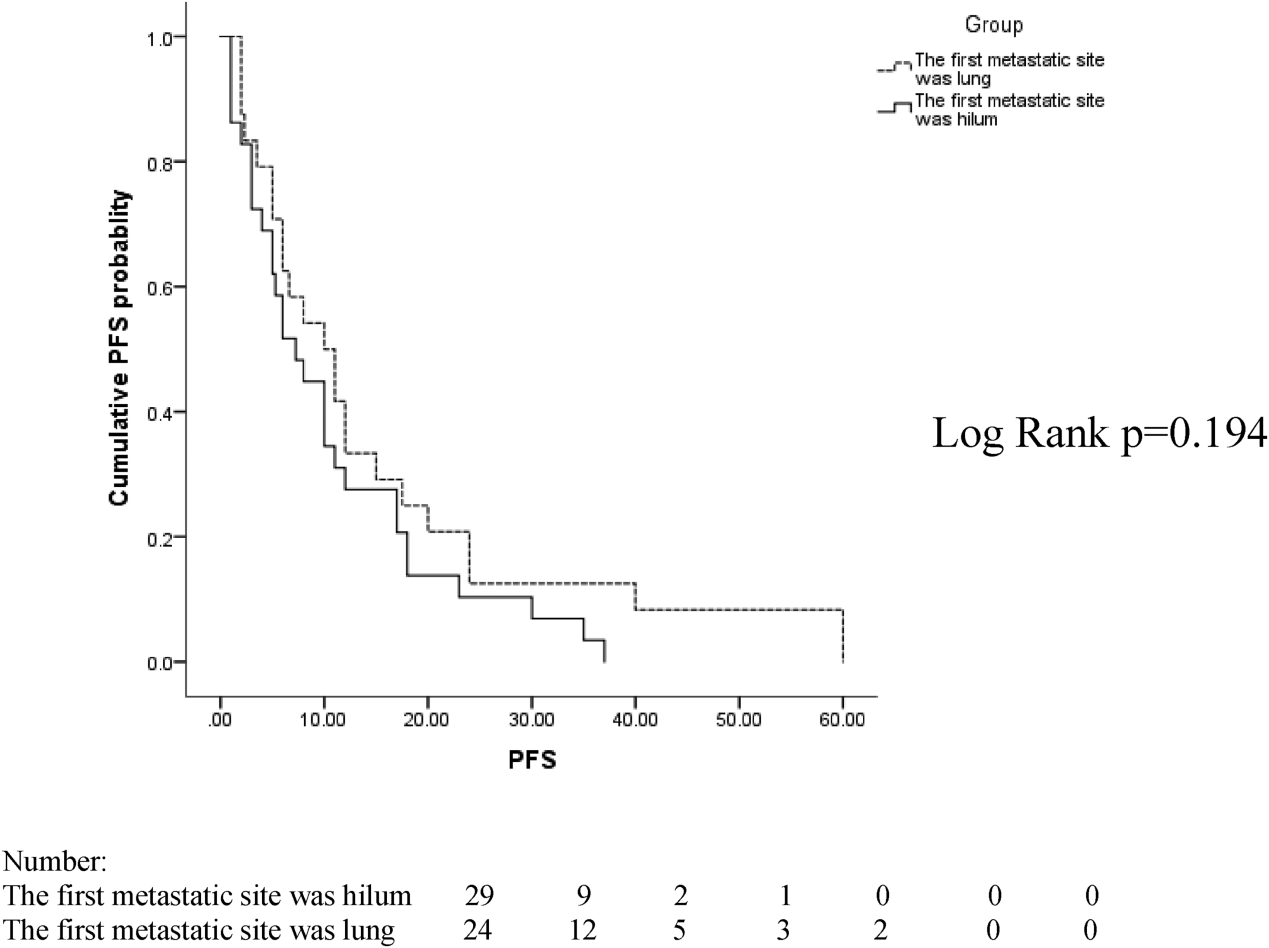
Progression-free survival (PFS) curves of patients with lung and hilar regions as the first metastatic sites.

**Figure 4:**
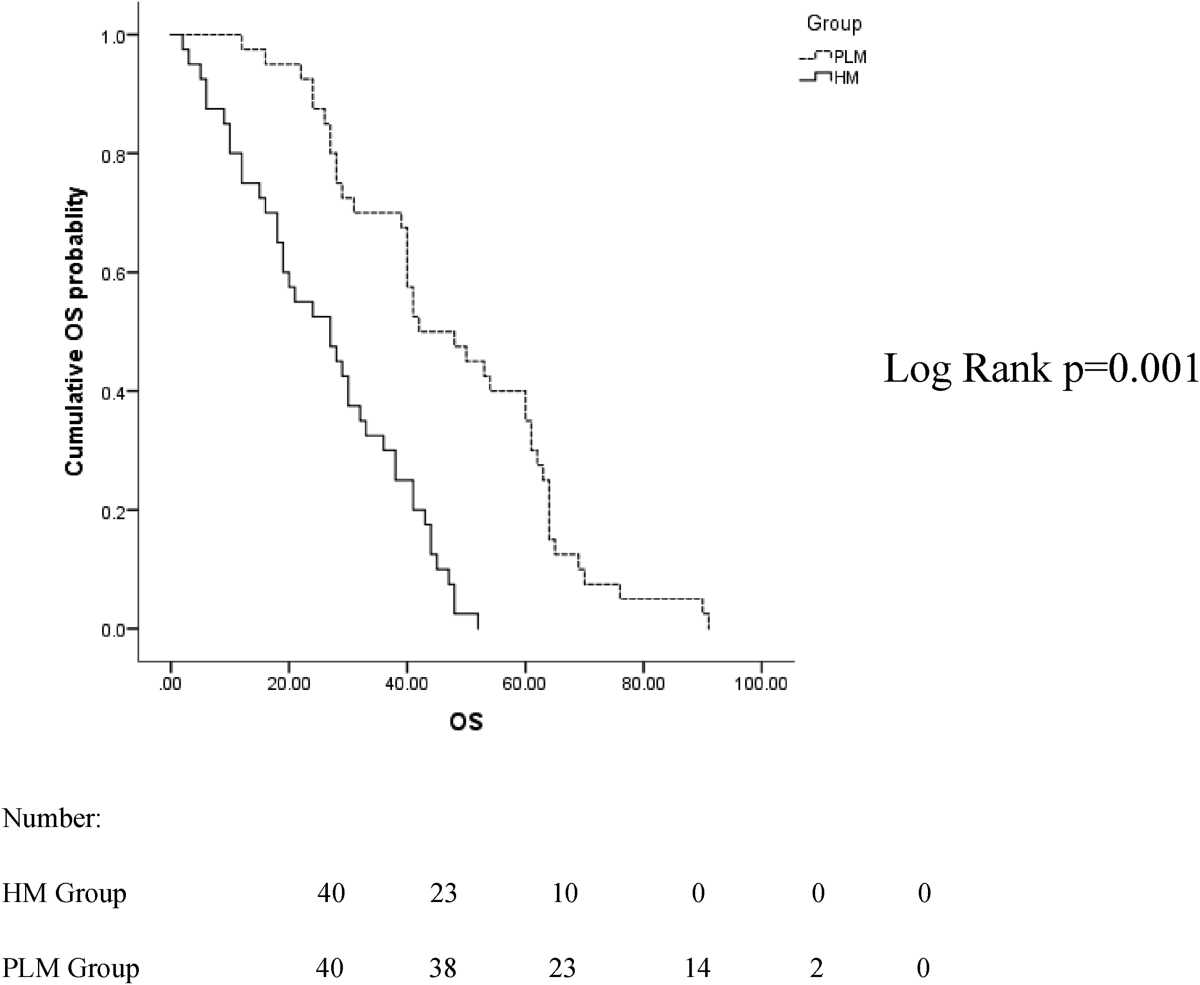
Overall survival (OS) curves for all patients in the hilar metastases (HM) and peripheral lung metastases (PLM) groups.

The median follow-up time between the two groups was 38 months, ranging from 2 to 91 months. In the two groups with the hilum (29 cases) and lung (24 cases) as the first metastatic site, we used the Kaplan–Meier method to draw the survival curves and analyze the differences in PFS and OS. The median PFS (mPFS) was 7.2 months for patients with the hilar region as the first metastatic site (with an mOS of 20) and 10 months for patients with the lung as the first metastatic site (with an mOS of 40). Further details are provided in Figures 2, 3. The Kaplan–Meier method drew the OS curve for all patients. The results showed that the mOS of patients in the HM group was 27, and that of the PLM group was 42 as shown in Figure 4. We also analyzed the relationship between various factors such as age, Ki-67 index, histological grade, molecular type, and the prognosis of the HM group. We found that only histological grade was associated with the prognosis of patients in the HM group (HR = 1.734, 95% CI 1.231–2.443, *p* = 0.002), as detailed in Table 5. We analyzed the relationship between histological grading and Ki-67 expression in both groups of patients (Table 6). The results showed no significant correlation between Ki-67 expression and histological grading in both groups of patients (*p* > 0.05).

**Table 5:** Multivariate Cox-regression of overall survival from time of hilar metastases.

Factor	β value	SE value	HR value	*p* value	95% CI	
					Lower	Upper
Age	−0.003	0.445	0.997	0.995	0.417	2.385
Molecular typing	0.379	0.271	1.460	0.162	0.859	2.481
Ki-67 expression	−0.187	0.593	0.829	0.752	0.259	2.651
Histological grading	1.008	0.328	2.741	0.002	1.442	5.208
Mediastinal lymph node metastasis	0.229	0.411	1.257	0.578	0.562	2.810
First metastatic site	−0.280	0.192	0.755	0.143	0.519	1.100
Whether the first episode is visceral metastasis	−0.074	0.607	0.929	0.903	0.283	3.052
Disease-free interval	0.321	0.482	1.378	0.505	0.536	3.544

**Table 6:** Relationship between histological grading and ki67 expression in two groups of patients.

Ki-67 expression	Hilar metastases group (*n* = 40)			Peripheral lung metastases group (*n* = 40)		
Histological grading	<15%	≥15%	*p*	<15%	≥15%	*p*
Grade I	0 (0)	6 (15%)	0.246	4 (10%)	12 (30%)	0.270
Grade II	3 (7.5%)	12 (30%)		7 (17.5%)	7 (17.5%)	
Grade III	1 (2.5%)	18 (45%)		2 (5%)	8 (20%)	

## Discussion

Breast cancer with HM as the primary manifestation is infrequent in clinical practice. Krutchik et al. reported an incidence of approximately 2%–5% (15, 20). Currently, the mechanism of metastasis is still unclear, and some studies suggest that endobronchial metastasis may be caused by bronchial mucosal metastasis of the tumor. Possible mechanisms include (1) direct metastasis to the bronchus, (2) bronchial invasion by a parenchymal lesion, (3) bronchial invasion by mediastinal or hilar lymph node metastasis, and (4) peripheral lesions extending along the proximal bronchus. Direct bronchial metastasis is the most common form of hilar metastasis (9, 10).

We reviewed all current cases reporting breast cancer hilar metastasis. Through literature, analysis found that the median age of patients with hilar metastasis was 54 years (33–72 years), of which 20 cases recorded the age of onset, with a total of four cases with ages less than 40 years. This is similar to the patients’ median age of 56 years (25–75 years) in our study with HM (14, 15, 20, 21). Although the median age was similar, nine (22.5%) patients in the HM group were younger than 40 years of age compared to none in the PLM group. The proportion of younger patients in the HM group was much higher than that in the PLM group, indicating that younger age may be a feature of the population with breast cancer HM. We analyzed the relationship between molecular typing and hilar metastasis; however, no molecular type was associated with hilar metastasis in the HM group. Luminal A and luminal B were more common, but the difference was not statistically significant. This is consistent with the results of the reviewed literature (13, 21, 22, 23). Further studies with larger sample sizes are warranted. No studies have reported the Ki-67 index or histological grade. In this study, it was found that the HM group had a significantly higher number of patients with histological grade III (19 versus 10, *p* = 0.025) and high Ki-67 expression (36 versus 26, *p* = 0.007) than the PLM group, indicating that the tumor was highly invasive. This finding is consistent with the poor prognosis of patients in the HM group, as described later. We found that the length of the DFI was inconsistent in patients with breast cancer HM. In seven cases reported by Ikemura et al. and Luo et al., one case had a DFI shorter than 12 months, which was 8 months, and six cases had a DFI longer than 12 months, which could reach a maximum of 336 months (13, 18). Our study found that 21 patients (52.5%) with HM had a DFI shorter than 12 months, more significant than the proportion in the PLM group (10 patients, 25%) and the reported cases. Twenty-four patients (60%) in the HM group had mediastinal lymph node metastasis, which was significantly greater than that in the PLM group (10 patients, 25%). We speculate that hilar metastasis may be achieved by mediastinal lymph node metastasis invading the bronchial mucosa.

Patients with HM present with hilar masses on imaging, showing burr and lobulation signs, obstructive pneumonia, and mediastinal lymphadenopathy. Bronchoscopic findings are mostly endobronchial masses, with a small proportion of patients presenting with bronchial wall thickening or bronchial mucosal edema (20). In our study, 40 patients in the HM group presented with a hilar mass on imaging, which is consistent with previously reported findings (24). Bronchoscopy did not reveal bronchial wall thickening or mucosal edema in any patients. The clinical symptoms and imaging features of patients with HM are consistent with the characteristics of primary central lung cancer or other hilar tumors and are non-specific; therefore, they cannot be used as a reference for differential diagnosis. Bronchoscopy is the primary clinical diagnosis.

In patients whose first metastatic sites were the hilar region, the mPFS was 7.2 months, which was of shorter duration than it that for the first metastatic site in the lungs (10 months). The mOS was 20 months shorter than that of patients whose initial metastatic site was the lungs (40 months), indicating that the prognosis of patients with the hilum as the first metastatic site was poor. To date, there have been no relevant reports found in the literature.

The mean mOS was 27 months in the HM group and 42 months in the PLM group. In 2019, Wang et al. reported that in 2005 breast cancer patients with PLM, the mOS was approximately 36 months, which is similar to the results of our study. In that study, the clinical characteristics of lung metastases were also analyzed. The molecular classification was more common in the luminal type and a low number of histological grade III patients, similar to the clinical characteristics of 40 patients in the PLM group. However, the median age of the patients with pulmonary metastases in that study was 66 years (25), higher than the median age of 59 years in the PLM group in our study, which may be related to the small sample size.

We performed Cox proportional hazards model analysis of multiple factors, such as age, menstrual status, Ki-67 index, and molecular classification. We found that histological grade III was associated with the prognosis of patients in the HM group, while other factors were not. Factors related to prognoses such as histological grading and ki-67 expression did not correlate in the two groups of patients. This may require expanding the sample size to confirm the correlation between other factors and the prognosis of breast cancer hilar metastasis.

## Conclusion

Hilar metastasis is a distinct form of distant metastasis of breast cancer. With a young age of onset, the histological grade and Ki-67 index are high, and the imaging manifestations include a single hilar burr-like or lobulated mass with mediastinal lymph node metastasis. This form of metastasis is highly invasive, with a shorter DFI and OS, resulting in the need for clinicians to be more flexible and individualize treatment options. This special mode of hilar metastasis should be considered when patients with breast cancer have the above-mentioned specific manifestations.

## Data Availability

The raw data supporting the conclusions of this article will be made available by the authors, without undue reservation.
